# Sensory and Physicochemical Evaluation of Acacia and Linden Honey Adulterated with Sugar Syrup

**DOI:** 10.3390/s20174845

**Published:** 2020-08-27

**Authors:** Zsanett Bodor, Zoltan Kovacs, Mahmoud Said Rashed, Zoltán Kókai, István Dalmadi, Csilla Benedek

**Affiliations:** 1 Department of Physics and Control, Faculty of Food Science, Szent István University, HU-1118 Budapest, Hungary; Bodor.Zsanett@hallgato.uni-szie.hu (Z.B.); kovacs.zoltan3@etk.szie.hu (Z.K.); mahmoud.said@alexu.edu.eg (M.S.R.); 2 Department of Postharvest Science, Trade and Sensory Evaluation, Faculty of Food Science, Szent István University, HU-1118 Budapest, Hungary; kokai.zoltan@etk.szie.hu; 3 Department of Refrigeration and Livestocks’ Products Technology, Faculty of Food Science, Szent István University, HU-1118 Budapest, Hungary; dalmadi.istvan@etk.szie.hu; 4 Department of Dietetics and Nutrition, Faculty of Health Sciences, Semmelweis University, HU-1088 Budapest, Hungary

**Keywords:** honey, electronic nose, electronic tongue, sensory analysis, adulteration

## Abstract

Honey is produced by honeybees and is used as a food and medical product. Adulteration of honey has been a problem for several years now because of the relatively high price of honey on the market according to its valuable composition. The aim of our study is to determine the physicochemical properties of authentic Hungarian linden and acacia honeys (pure samples or manipulated ones blended with sugar syrup) as well as commercially available blends of European Union (EU) non-European Union (non-EU) honeys. Authentic linden and acacia were blended with sugar syrup at 10%, 20% and 50% concentration levels, and physicochemical properties were determined according to the methods of the International Honey Commission. Our objectives also included testing of the performance of electronic sensory techniques (electronic tongue (ET) and electronic nose (EN)) in the detection of adulteration, and the results are compared to the sensory profile analysis. The results provide good average recognition and prediction abilities for the classification of adulterated and authentic honeys (>90% for ET and higher than >80 for EN). Misclassifications were found only in the case of honey with 10% added sugar syrup. The methods were also able to reveal adulteration of independently predicted samples.

## 1. Introduction

Honey is a supersaturated sugar solution produced by honeybees *(Apis mellifera)*. Its two main sugars are glucose and fructose, but other di- and trisaccharides are also present [1]. Depending on their botanical and geographical origin, vitamins, minerals, phenolic acids, flavonoids, organic acids and amino acids are present in honey in different amounts [2,3,4,5,6,7,8]. Origin also has an effect on the sensory profile of honeys [9,10,11]. Both composition and sensory attributes can be influenced by processing and storage conditions [12]. In recent years, honey adulteration has increased significantly because of its relatively high market price and its popularity due to its valuable nutritional content. Fraud related to honey include several procedures, e.g., feeding bees with different sugar syrups (indirect method) and blending honeys with the syrups (direct method). Depending on the plant source, the syrups used for honey adulteration are either C3 (e.g., rice, beet and wheat) or C4 (e.g., corn and cane) sugars. Further known types of adulteration are mislabelling honeys as coming from a different geographical location, application of heat treatment for elimination of naturally formed crystals or dyeing honey with sulphite-ammonia caramel to get more favourable colours [13].

Fraud detection related to the botanical or geographical origin of honey is a challenging task due to the complex composition of honey. Promising but time-consuming, destructive and expensive instrumental analyses are used by researchers and authorities to check the authenticity of honey, e.g., nuclear magnetic resonance (NMR), isotopic ratio mass spectrometry coupled with elemental analysis and liquid chromatography (EA/LC-IR-MS), or three-dimensional fluorescence spectroscopy [14]. However, pollen analysis combined with physicochemical properties and sensory analysis are the most commonly used techniques for identifying the botanical origin of honey [15]. Detection of certain adulterations of honey is also possible with the physicochemical and colour attributes of honeys. Amiry et al. (2017) used physical, rheological, colour and chemical parameters of honey to build models for the detection of honey containing invert sugar syrup at 7%, 14% and 30%. Physical and chemical parameters provided promising results based on the linear discriminant analysis models built [14]. Turkish researchers analysed natural honey mixed with fructose and sucrose syrup at the 10%, 20%, 30%, 40% and 50% levels. The decreasing tendency in pH, ash content, a* (redness) and b* (yellowness) were found to be in an inverse correlation with the increasing adulteration level, while L* (lightness) increased [16]. In a Brazilian study, blossom honey was adulterated with high fructose content corn syrup at the 10%, 25%, 50%, 75% and 100% levels. In this study, increasing pH, L* and moisture content while decreasing b* were found as adulteration level increased [17].

In previous studies, sensory parameters were used for the detection honey adulteration by both lay consumers and trained panels. Brazilian researchers adulterated citrus honey with glucose syrup at the 20% and 50% levels. Consistency, the specific smell, sweet taste and colour of honeys were compared. Based on the scores given by the consumers, the consistency of the samples was found to be different [18]. Guler et al. (2008) tested the sensory properties of honey adulterated by feeding bees with sucrose syrup. For filtered honeys, significant differences were found in odour, flavour and taste, while in comb honeys, there were no significant differences between the control and adulterated honeys [19]. These studies show that sensory evaluation is applicable for the detection of adulterated honey. However, this technique is usually used for discrimination between honeys from different botanical origins [20]. Instrumental sensory analyses, such as the electronic nose (EN) or electronic tongue (ET), can also be successfully applied for testing the authenticity of food, including bee products [21]. Several studies report the application of the electronic tongue and nose for the identification of origin and for the detection of adulteration of honey. Malaysian researchers used both ET and EN and combined these techniques to identify the botanical origin of honey and to discriminate honey samples adulterated with sugar syrup. Linear discriminant analysis (LDA) models provided higher than 90% classification accuracy when ET and EN methods were applied separately, while 100% accuracy was reached by the fusion of the data of both techniques [22]. In a Spanish research, a pulse voltammetric electronic tongue was applied with Principal Component Analysis (PCA) to distinguish between honey samples adulterated at different levels by using different syrups. Results showed good separation not only for syrups and honey types but also between pure honey and adulterants, and Partial Least Square (PLS) models were able to predict the concentration and type of adulterant [23]. Oroian et al. [24] also applied a voltammetric electronic tongue for the detection of adulteration of honey containing different syrups at the 0–50% levels of adulteration. The electronic tongue achieved an 83.33% correct classification in the differentiation of honeys adulterated with different syrups, while 97.56% was achieved for pure honey and 100% was achieved for correct classification of adulterated honey. 

It can be concluded that the electronic tongue, electronic nose and sensory analysis have been used successfully to detect the adulteration of honey. It is important to mention that mostly voltammetric electronic tongue techniques were used. Moreover, the three methods, sensory profile analysis, electronic tongue and electronic nose, have not been applied together for the detection of the honey adulteration. Finally, they have not been used to predict the authenticity of blended honeys of both EU and non-EU origins.

Therefore, in this study, our objective is to determine the physicochemical and sensory properties of acacia and linden honeys pure or with added sugar syrup and to analyse the ability of electronic tongues and electronic noses compared to sensory profile analysis in the detection of adulteration of the aforementioned honeys. Moreover, our goal was to build a model able to predict the authenticity of blended honeys originating from both European Union and non-European Union countries.

## 2. Materials and Methods

### 2.1. Samples

Acacia (*Robinia pseudoacacia)* and linden (*Tilia* spp.) honeys were analysed in our study: 22 acacia and 12 linden honeys were collected in total. Of these, 10 acacia and 3 linden honeys were purchased from retail and labelled as blends of European Union and non-European Union honeys (from here on, EUnonEU acacia and EUnonEU linden) in accordance with the European Council (EC) legislation [25]. The EUnonEU honey blends are coded according to their honey type and registration number in our database (e.g., HL_98 for linden honey and HA_100 for acacia honey). The authentic samples (11 acacia and 9 linden) were collected directly from beekeepers. The geographical origins of the authentic samples can be seen in Table 1.

Sugar syrup (glucose-fructose) was mixed with an authentic acacia and an authentic linden honey in concentrations of 10% (1:9), 20% (1:4) and 50% (1:1), resulting in three model adulterated samples for each botanical type. These are coded as A10, A20 and A50 and as L10, L20 and L50, where “A” is for acacia and “L” is for linden. The numbers denote the concentration of sugar syrup in %.

### 2.2. Methods

#### 2.2.1. Reference Methods

##### Physicochemical Indicators

The physicochemical indicators of honey samples (ash content, electrical conductivity, pH and total soluble dry matter (TSDM%) by refractometry) were determined according to the International Honey Commission method book [26]. Each sample was measured in three replicates, resulting in 120 observations.

##### Sample Preparation for Antioxidant Capacity Assays

Sample preparation for each method was the same: 1.0 g of honey was weighed on an analytical scale, then dissolved in distilled water and filled up to 10 mL in volumetric flasks. Each sample was measured in five replicates, resulting in 200 observations per each parameter.

##### Total Polyphenol Content (TPC)

Total polyphenol content was measured by the Folin–Ciocalteau method [27]. Of the honey sample solution, 1 mL was put in a test tube and 7.5 mL distilled water was added. Then, 0.5 mL of the Folin–Ciocalteu reagent was given to each tube, and after 3 min, 1 mL Na_2_CO_3_ solution was added. Absorbance values were read at 750 nm with a Helios α-spectrophotometer after 30 min of incubation in the dark. Gallic acid was used as a calibration standard.

##### Ferric Reduction Antioxidant Power (FRAP)

As a first step of this method, as described by Benzie and Strain [28], the FRAP reagent was prepared: 0.54 g FeCl_3_ was measured in a 100 mL volumetric flask and dissolved up to volume with distilled water, and 0.3123 g 2,4,6-tripyridyl-S-triazine (TPTZ) was dissolved up to 100 mL with 40 mM HCl. The two solutions were mixed with 500 mL acetate buffer at pH 3.6. Following the preparation of the reagents, 500 µL of the honey sample was pipetted in a test tube and then 7.5 mL of the FRAP reagent solution was added. This solution was incubated at 37 °C for an hour and then measured at 653 nm. Ascorbic acid was used as a calibration standard.

##### Cupric Ion Reducing Antioxidant Capacity (CUPRAC)

The procedure was developed by Apak et al. [29]. For the measurement, 1 mL of CuCl_2_ (10^−2^ M), 1 mL of NH_4_-acetate buffer solution (pH = 7), 1 mL of neocuproine solution (0.156 g neocuproine in 100 mL ethanolic solution), 200 µL of the honey sample solution and 0.9 mL distilled water were mixed. After 30 min of incubation in the dark, the solutions were measured at 450 nm. Trolox was used as a calibration standard.

##### Sugar Determination by HPLC

The sugar composition (fructose, glucose and saccharose) was determined by RP-HPLC (Waters, Milford, Massachusetts, USA), with a refraction index detector, using the Kromasil 100-5 NH_2_ MZ column (250 mm × 4.6 mm, particle size of 5 µm). The flow rate was 1.5 mL/min, the detection was performed at 25 °C, and the mobile phase was 28:72 *v/v* water acetonitrile solution.

Glucose, fructose and saccharose standards were prepared at three different concentration levels for the calibration. Honey samples were prepared in two replicates: 1 g honey was dissolved in analytically pure distilled water, then transferred to a 100 mL volumetric flask and filled up to volume. These solutions were filtered through a Chromafil XTRA RC45/24 filter, and then a 10 µL sample was injected.

##### Colorimetric Measurement

Determination of colorimetric properties of different honeys was performed in the CIE (International Commission on Illumination) L*a*b* tristimulus coordinate system with Konica Minolta 410 colorimeter in five replicates per sample, resulting in 200 observations. L* is assigned to the lightness (0–100, higher L* values denote lighter samples), a* values (−50–+50) are assigned to the greenish (negative direction) or reddish (positive direction) hue, while b* values (−50–+50) are assigned to the blueish (negative direction) or yellowish (positive direction) hue.

#### 2.2.2. Sensory Profile Analysis

The sensory profile analysis of honeys was performed in a sensory laboratory, fulfilling the requirements of the relevant International Organization for Standardization (ISO) standards [30,31,32]. The sensory panel consisted of 12 members. In the sensory test, six acacia and six linden honeys were tested; for each floral type, two authentic honeys were used as references. Samples included one EUnonEU honey (HA_100 and HL_98 honeys) and three honey samples with added sugar syrup (10%, 20% and 50% sugar syrup content). The two honey types (acacia and linden) were tested in two different sessions. Each honey type was tested in two independent sessions by the same sensory panel. Honeys were examined based on odour and taste/flavour characteristics, with 13–13 properties per each honey type shown in Table 2. The sensory attributes were chosen from the honey aroma wheel [20]. Honeys were diluted with water in a 4:1 ratio for better sensory differentiation.

#### 2.2.3. Electronic Tongue (ET)

Electronic tongue (ET) measurements were performed by an αAstree electronic tongue [33] which was designed to recognize and analyse the dissolved compounds in liquid samples. The ET consists of a sensor array with seven potentiometric CHEMFET (chemically modified field effect transistor) sensors developed for food applications and an Ag/AgCl reference electrode. During the measurement, the potential difference is recorded between the reference electrode and the individual working electrodes which depends on the chemical composition of the sample, providing a unique fingerprint of the tested liquid samples. A tenfold dilution was prepared for the electronic tongue measurement: 10.0 g honey was weighted in and filled up to volume in a 100 mL volumetric flask. Three replicate honey samples were tested in repeated measurements on three different days, resulting in 9 repetitions per day. Honeys which were used for the sensory profile analysis were tested in two replicates on two different days. For each measurement day, two reference sample were measured to be able to correct the drift of the different days as a result of ageing of the sensors. After outlier detection, 447 observations were acquired for acacia and 207 were acquired for linden honeys.

#### 2.2.4. Electronic Nose (EN)

Electronic nose measurements were performed using an NST3320 type electronic nose (Applied Sensor A.G., Linköping, Sweden) with a built-in headspace autosampler for 12 samples. In the sample chamber, 23 different sensors can be found: 10 MOS FET (metal oxide semiconductor field effect transistor) sensors, 12 MOS (metal oxide semiconductor) sensors, and a sensor for humidity acquisition. As a reference gas, ambient air was used, which was filtered through a silica gel column and a moisture/hydrocarbon filter. The gas flow rate of the dynamic sampling was set to 50 mL/min. The sequence of EN measurements began with the equilibration of the sample at 30 °C for 15 min. Then, the reference air was pumped over the sensor surfaces for 10 s (baseline), followed by the honey head space for 30 s (sampling time), while the sensor signals were recorded. The sample analysis was followed by the recovery phase which was set to 260 s including the flush time of the gas lines with the filtered air prior to the next sample injection allowing reestablishment of the baseline of the instrument. Altogether, the total cycle time was 500 s. Each honey sample was measured three times with three consecutive measurements, resulting in nine replicates per sample. After outlier detection, 162 observations were left for acacia honeys and 115 were left for linden honeys. 

#### 2.2.5. Statistics

Statistical evaluation of data was performed by descriptive statistics for the results of the methods mentioned in the Reference Methods Section 2.2.1 normality of the residuals was tested with the Shapiro–Wilk test followed by ANOVA evaluations with either the post hoc Tukey test or Games–Howell test. For the latter, homogeneity of variances was not assumed based on the results of the Levene test for the detection of significant differences between determined groups [34]. Significant differences among the groups of EUnonEU, authentic and adulterated honeys were analysed. Moreover, ANOVA models were also built to check the significant differences among authentic acacia and some selected individual EUnonEU blends from both types of honey. In the case of linden honey, all EUnonEU honeys (HL_79, HL_83 and HL_98) were used for the ANOVA test. In the case of acacia honey, four samples were chosen (HA_78, HA_84, HA_99 and HA_100) based on the results of the independent prediction, in order to have samples from suspected adulterants and non-adulterants.

Multivariate statistics like principal component analysis (PCA) and linear discriminant analysis (LDA) were applied on the results of ET and EN. PCA was used for the identification of outliers as an exploratory data evaluation method and for visualization of the main patterns and information of the multivariate sensor set. Before the analyses, in the case of electronic tongue results, a drift correction (additive correction relative to reference samples) was used for correcting the drift between different measurement days [35]. LDA classification models were built for the two honey types separately. Models were built for the classification of authentic honeys (non-adulterated, collected from beekeepers) and the adulterated ones by 10%, 20% and 50%. For the chosen EUnonEU honeys, an independent prediction was applied on the built classification models to detect which one classified as authentic honey or else as a sugar syrup blend honey to be able to exclude the suspicion of adulteration. LDA models and an independent prediction were built with a three-fold-cross-validation. Partial least square regression (PLSR) with leave-one out cross validation (LOO) was applied to predict the sensory properties of the acacia and linden honeys using the results of the electronic tongue and nose separately. The parameters provided significant differences between on the one hand the authentic and adulterated honeys and between the authentic and EUnonEU honeys on the other. The regression error was analysed using root mean square error (RSMEC) in the case of training and using RMSECV in the case of validation. The sensory properties of the chosen EUnonEU samples (HA_99, HA_78, HA_84, HL_79 and HL_83) were predicted using the PLSR prediction model built for the 6–6 samples (the samples which were tested in the sensory profile analysis) based on the data of the electronic nose and tongue separately. R-project 3.5.2, SPSS 25 and Microsoft Excel software were used for statistical analysis.

## 3. Results

### 3.1. Results of the Reference Methods

The results of the reference methods for acacia and linden honeys can be found in Table 3. Physicochemical indicators such as total soluble dry matter, pH and electrical conductivity showed lower values for the adulterated samples compared to authentic honeys in the case of acacia honeys. For linden honeys, electrical conductivity showed higher results compared to authentic honeys, but a decreasing tendency was noticed by the increase of syrup concentration. The higher values in adulterated honey can be explained by the fact that, on the one hand, authentic honeys have high standard deviation and, on the other hand, the honey that was diluted with the syrup had high electrical conductivity (681 ± 1.87 µS/cm) itself. Adulterated honeys had significantly lower total soluble dry matter (TSDM%) content compared to authentic honeys in both honey types. ANOVA results of the chosen individual honey types (Table 4) showed significantly higher total soluble dry matter content in the case of HA_100 and HA_84 honeys, while HA_78 had significantly lower TSDM% compared to authentic acacia honeys. Compared to the authentic honeys, the results of linden honey revealed insignificant differences in both TSDM% and pH.

The results of the sugar composition showed (Table 3) that authentic linden and acacia honeys had significantly lower glucose and fructose contents compared to the EUnonEU honeys; however, fructose/glucose ratios were similar to the fructose glucose ratio determined by the International Honey Commission (IHC): authentic acacia 1.66 ± 0.14, EUnonEU acacia 1.61 ± 0.22, authentic linden 1.31 ± 0.14 and EUnonEU linden 1.19 ± 0.08 [36].

Colour evaluation of honey (Table 3) provided a similar tendency for linden and acacia honeys. The L* value of adulterated honey was significantly higher, with dilution resulting in lighter honeys, while a* and b* were significantly lower, resulting in greener and less yellowish honey blends. These results are in accordance with the results of Brazilian and Turkish studies [16,17].

The results of antioxidant capacity measurements of acacia honeys showed a decreased capacity with an increased ratio of sugar syrup (Table 3). In the case of acacia honeys, A50 had a significantly lower total polyphenol content than authentic acacia, EUnonEU acacia and A10. The results of the antioxidant capacity measurements showed that A50 had significantly lower CUPRAC and FRAP values compared to the other groups. Significantly lower CUPRAC results were obtained for EUnonEU honeys compared to authentic honeys. A10 and A20 also resulted in significantly lower FRAP values compared to authentic honey and EUnonEU acacia honeys. The results of the ANOVA test for the chosen commercial EUnonEU (Table 4) honeys showed that HA_100 and HA_99 honeys had significantly lower antioxidant capacity compared to authentic honeys. There was no significant difference in the total polyphenol content (TPC). HA_78 had significantly higher FRAP, albeit not significantly lower CUPRAC values. The linden honey results showed significantly lower TPC for L50 honey compared to authentic, L10 and L20, while there was no significant difference between L50 and EUnonEU honeys. A significantly lower antioxidant capacity was found in the L50 and EUnonEU linden honeys in comparison with authentic honey. Individual EUnonEU linden honey results (Table 4) revealed significantly lower FRAP and CUPRAC scores in the case of HL_83 and HL_98, while HL_79 had significantly lower FRAP results compared to authentic linden honey.

### 3.2. Results of the Sensory Profile Analysis

**Figure 1:**
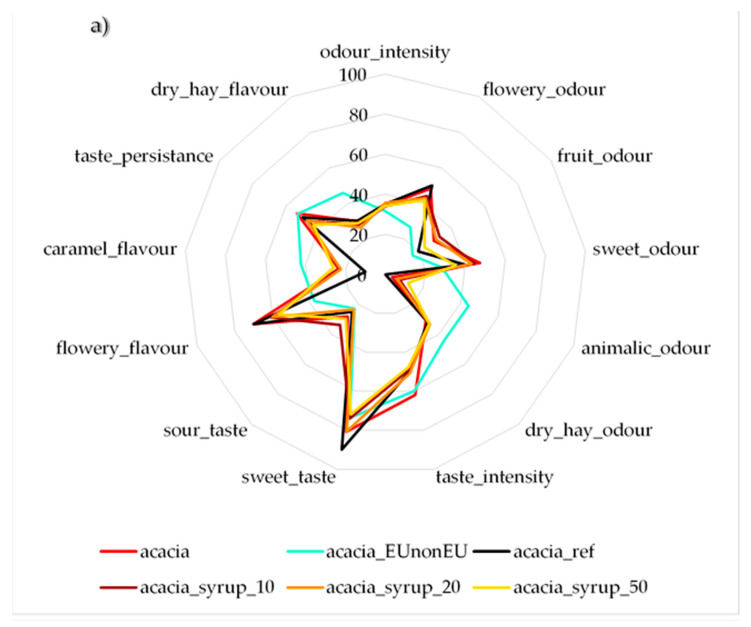

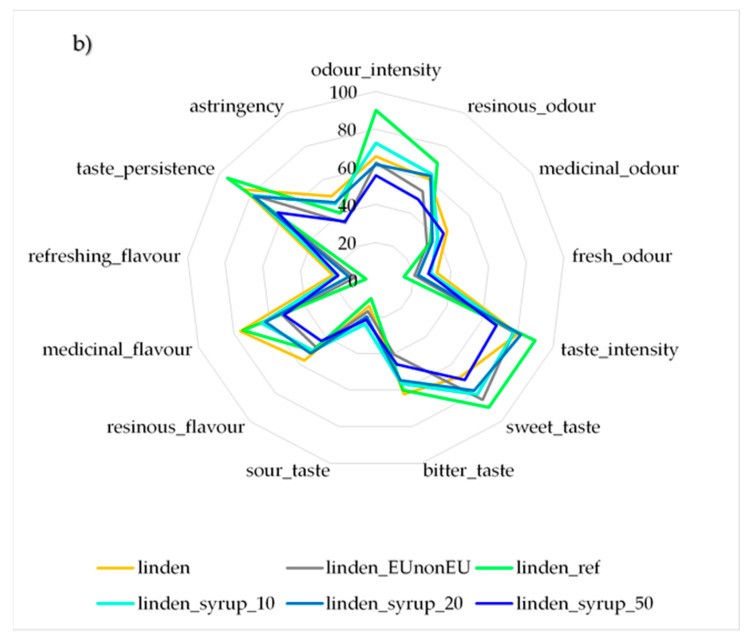
Results of the sensory profile analysis of (**a**) acacia and (**b**) linden honey, *n* = 12/parameter.

The sensory profile of adulterated and authentic acacia honeys is shown in Figure 1a. The evaluation revealed significant differences between the reference and adulterated honeys in four parameters: A10 honey reached a significantly higher score for fruity odour, A10 and A50 received significantly lower sweet taste and flowery taste scores, while A20 and A50 had significantly higher caramel taste scores compared to reference honey (*p* < 0.05), based on results of ANOVA test followed by the Games–Howell post hoc test. However, other parameters showed differences that were not statistically significant: adulterated honeys had lower odour scores (odour intensity and fruity odour) and higher values in sweet odour, animalic odour, fruit odour and dry hay odour. Taste parameters showed lower values in the case of intensity, sweetness, flowery, dry hay taste and taste persistence. Higher values were obtained for caramel flavour and sour taste. The EUnonEU acacia honey showed significant differences from at least one of the authentic honeys in flowery, sweet and animalic odour and in sweet, flowery and caramel taste. Sugar composition analysis showed that the EUnonEU honey blend had higher glucose and fructose contents (312.41 g/kg and 503.42 g/kg) than the reference honey (285.04 g/kg and 414.64 g/kg), which can explain of the sweeter taste and odour perceived.

The results of the linden honey sensory evaluation (Figure 1b) showed significant differences between reference and adulterated honey for eight parameters (*p* < 0.05): L10 was significantly different in fresh odour; L20 was different in sour taste; L50 was different in bitter taste and medicinal flavour; and L10, L20 and L50 were different in taste and odour intensity and in sweet taste. Odour intensity, resinous odour, taste intensity, sweet, bitter, medicinal taste, astringency and taste persistence were weaker in adulterated samples, while refreshing and sour taste, and fresh and medicinal odour were stronger compared to the reference linden sample. The mixture of EU and non-EU originated honey showed significant differences from at least one of the authentic linden honeys in seven parameters: taste intensity and persistence, refreshing, medicinal and bitter taste, taste intensity, resinous odour and odour intensity. Turkish researchers found also significant differences in aroma, taste and odour between pure and adulterated honeys [19]. 

### 3.3. Results of Electronic Tongue Analysis 

**Figure 2:**
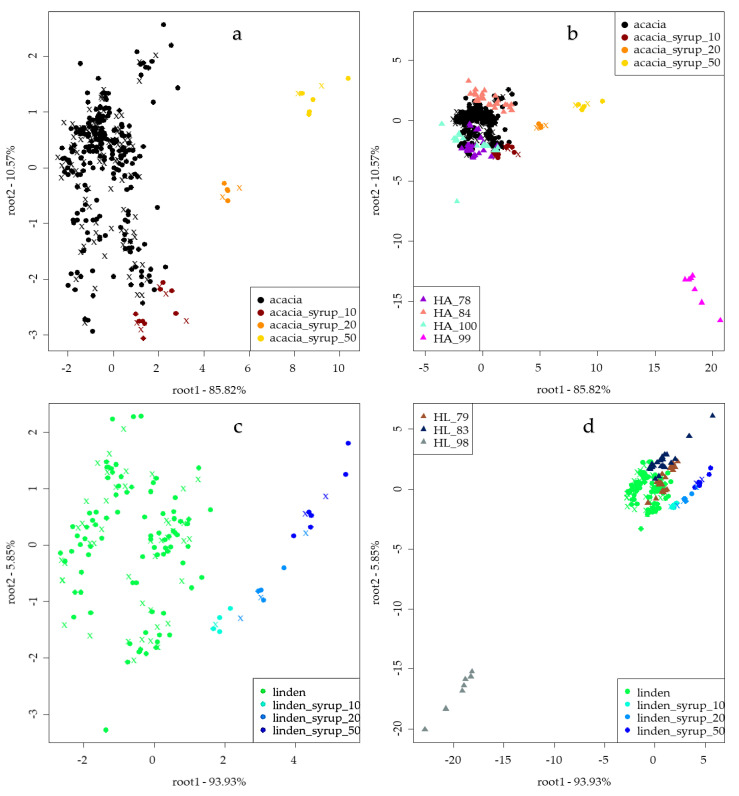
Results of electronic tongue linear discriminant analysis (LDA) models for the classification of authentic and adulterated acacia (**a**) (*n* = 381) and linden (**c**) (*n* = 148) honeys and independent prediction of EUnonEU blends for acacia (**b**) (*n* = 381 + 66) and linden (**d**) (*n* = 148 + 59) honeys; ● training, ✖ validation and ▲ independent prediction.

The LDA model built for the classification of authentic acacia and linden honeys based on the results of an electronic tongue can be seen in Figure 2. For both honey types, separation tendencies can be observed through root 1, according to the level of adulteration. Nevertheless, on root 2, separation of adulterants can be seen. The model of acacia honeys (Figure 2a) all presented average recognition and prediction abilities of 99.22%. Adulterated honeys (A10, A20 and A50) were classified correctly, while misclassification was found for authentic honeys belonging to A10 in 3.11%. Independent prediction of the four EUnonEU blend acacia honey samples (Figure 2b and Table 5) showed that honey HA_84 was correctly classified as authentic acacia honey. In the case of the HA_78 and HA_100 honeys, misclassification was found; they were classified to the A10 group in 3.85% and 11.76%, respectively. Honey HA_99 was classified as an adulterated honey belonging to group A10 and the A50 groups in 33.33% and 66.67%, respectively.

The LDA model of linden honeys (Figure 2c) presented average recognition and prediction abilities of 98.23% and 92.92%, respectively. Authentic linden, L10 and L50 honeys were classified correctly. L20 presented 92.92% and 71.67% correct classifications during training and validation. Misclassification was found in training; these resulted as belonging to L50 (7.08%). During validation, 14.14% was misclassified to L10 and L50. Independent predictions of the three EUnonEU blends (Figure 2 d and Table 5) showed that two of the honeys were misclassified as authentic Hungarian linden (HL_79 and HL_83), while HL_98 was classified as adulterated honey with 50% syrup content (L50).

### 3.4. Results of Electronic Nose Analysis

**Figure 3:**
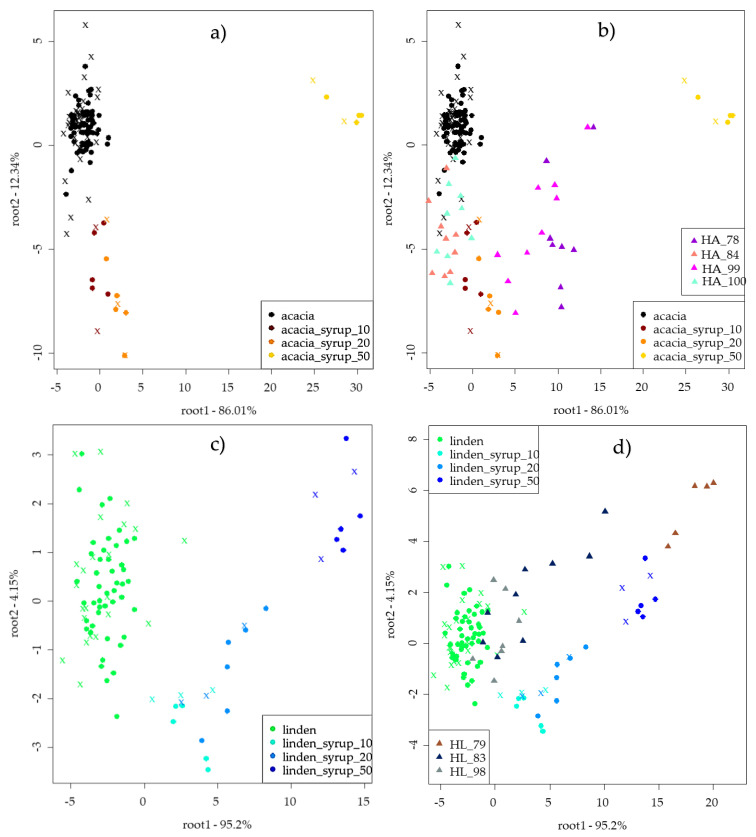
Results of the electronic nose LDA model for classification of authentic and adulterated acacia (**a**) (*n* = 127) and linden (**c**) (*n* = 94) honeys and models for independent prediction with EUnonEU blends for acacia (**b**) (*n* = 127 + 35) and linden (**d**) (*n* = 94 + 21) honeys; ● training, ✖ validation and ▲ independent prediction.

The LDA results of the electronic nose for classification of authentic and adulterated acacia and linden honeys with independent prediction can be seen in Figure 3. The LDA model of acacia presented a separation of A50 from other groups, while root 2 showed the tendency of separation between points of authentic as well as A10 and A20 honeys. The LDA model of acacia honey presented average recognition and prediction abilities of 95.31% and 88.77%, respectively. The training set provided correct classification of authentic acacia, A10 and A50 honeys; in the case of A20 honeys, misclassification was found belonging to A10 in 18.76%. The validation set revealed weaker results: A50 was classified correctly, misclassification was found for authentic acacia honeys belonging to A10 and A20 in 2.83%, A10 honey showed 85.84% of correct classification (misclassified as authentic honeys in 14.16%) and A20 honey showed misclassification to A10 in 25.09%. Independent prediction of the ten EUnonEU honeys showed that two of the honeys were classified as authentic, and others showed misclassification as A10, A20 and A50 honeys too. The results of detailed independent predictions can be seen in Table 5.

LDA results of linden honeys for classification of authentic and adulterated honeys based on the results of an electronic nose show a separation through root 1 (Figure 3c). The model provided average recognition and prediction abilities of 94.45% and 80.83%, respectively. Authentic linden, L10 and L50 were classified correctly in training, and misclassification was detected for L20, misclassified as L10 in 22.17%. Validation provided correct classification for L50, and 92.74% of authentic honeys were classified correctly (misclassification to L10 (4.35% and L20 2.91%). L10 was classified correctly in 74.91% (misclassified as L20), and 55.67% of L20 was classified correctly. Independent prediction of EUnonEU linden honeys presented misclassifications to adulterated groups (Figure 3d). One of the honeys was completely classified as counterfeit. Detailed classification can be found in Table 5.

Independent prediction of EUnonEU acacia honeys (Table 5) showed misclassification for HA_100 and HA_84 honeys to A10 honeys in 59.26% and 70.37%, respectively. HA_78 and HA_99 honeys were classified as 20% adulterated in 94.74% and 83.33%, respectively. Linden honey showed similar results, where HL_79 was classified as 50% adulterated.

### 3.5. Results of Partial Least Square Regression to Predict the Properties of Sensory Profile Analysis Using ET and EN

The results of the regression on the sensory properties using data of the electronic tongue and nose provided better results in the case of linden honey for both instruments; however, the results of the individual parameters were different depending on the device used (Table 6). In the case of the electronic tongue for the prediction of sensory properties of the acacia honey, the best prediction was obtained for animalic odour and flowery flavour with R^2^CV of 0.9415 and 0.888, respectively. Similarly, EN provided the best prediction for flowery flavour and sweet taste with values of R^2^CV of 0.4083 and 0.4578, respectively. Prediction of fruity odour using the results of the electronic nose was not achieved after cross-validation. In conclusion, the results showed that PLSR models of the electronic nose were weaker for all parameters compared to the results of the electronic tongue.

The same situation was noticed in the case of the results of linden honeys. However, prediction of the sensory parameters in this case provided better results compared to the results of the acacia models. PLSR models built using data of the electronic nose provided the best results for the prediction of the taste persistence, odour intensity and resinous odour with higher R^2^CV of 0.9. The electronic nose reached the highest performance in the prediction of medicinal flavour and bitter taste.

**Figure 4:**
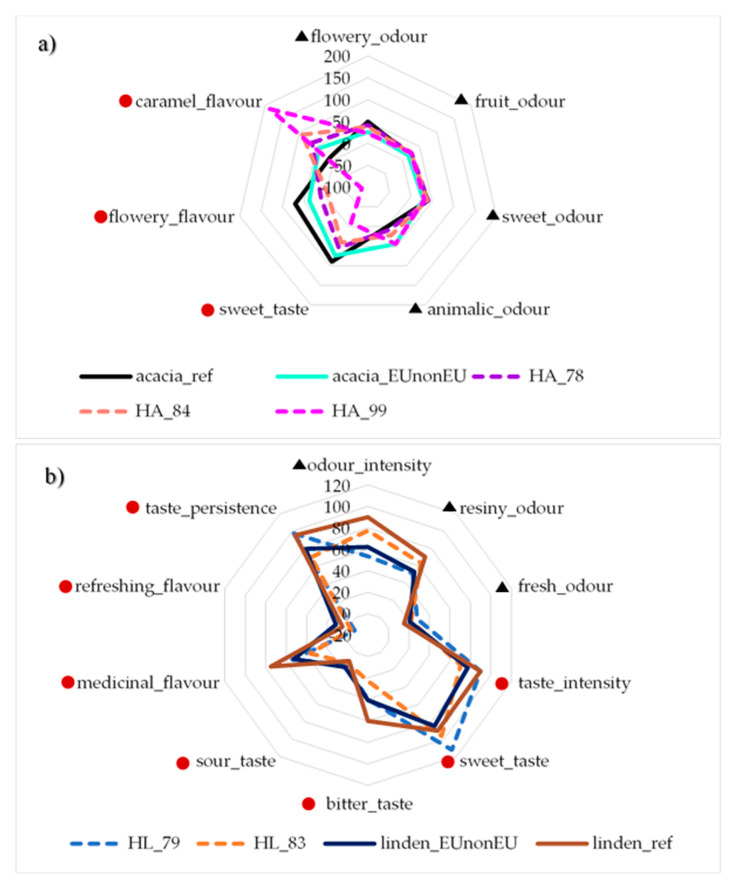
Results of the independent prediction of sensory parameters using the results of the electronic tongue and nose for (**a**) acacia and (**b**) linden honeys; 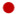 predicted from the results of ET and ▲ predicted from the results of EN.

Independent prediction of the sensory parameters of the acacia (HA_78, HA_84 and HA_99) and linden honey (HL_79 and HL_83) can be seen in Figure 4, where taste parameters were independently predicted using PLSR models of ET and odour proportion PLRS models of EN (Table 6). The results of acacia honey showed that HA_99 honey was very different from the reference honey. However, HA_84 and HA_78 were similar to each other and to the reference honey sample. The results of linden honey showed that the HL_83 and HL_79 honeys were sweeter and weaker in other aroma properties than the reference linden honey, similarly to the EUnonEU blend of linden honey (HL_98).

## 4. Discussion

The significantly lower total soluble dry matter content of the sugar syrup shows that lower contents (75.4%) affected the moisture content of the honeys (Table 3). These results are in accordance with other studies, where increased sugar syrup addition resulted in higher moisture (lower dry matter) content [16,17,37]. Significantly lower pH values of adulterated honeys can be explained by the more acidic sugar syrup (pH 3.54 ± 0.02). Similar results were obtained by Turkish [16] and Romanian researchers [38].

A decreasing tendency was found with the increase of added syrup concentration in total polyphenol content and antioxidant capacity measuring assays such as FRAP and CUPRAC (Table 3) that can be explained with the low antioxidant capacity of the sugar syrup. Turkish researchers also found a lower antioxidant capacity in uncertified honeys [39]; however, the lower values could be due to different geographical origins too.

Sensory profile analysis of the adulterated and unadulterated acacia and linden honeys revealed significant differences between authentic and adulterated honey. In the case of acacia honey, only fruity odour, caramel, flowery flavour and sweet taste showed significant differences between the authentic and adulterated honeys, while for linden honey, more parameters showed significant differences (Figure 1). This can be explained by the more pronounced aromatic properties of linden honey. Significant differences were also found comparing the EUnonEU honey blend with the authentic honeys for both acacia and linden. However, the two authentic honeys also differed in some properties. This can be explained by the different geographical origins and natural variability of the honey.

Electronic tongue (Figure 2) and electronic nose (Figure 3) measurements were more sensitive compared to sensory profile analysis. Adulterated honeys were discriminated from the authentic honeys; moreover, the authentic honey was classified correctly in the case of linden honey, and only 3.11% misclassification was found for acacia honeys (to the A10 honey) for the electronic tongue. Similar results were found in a Spanish study using an automatic pulse voltammetric electronic tongue, where honeys were adulterated with sugar syrup in 0–40% and PCA results showed that they could distinguish pure honey from adulterated ones [23]. A voltammetric electronic tongue was applied in a Romanian study, resulting in 97.56% and 100% correct classification of pure and adulterated honey with different syrups in 0–50%. This accuracy is also similar to the one obtained in our study [24]. Chinese researchers also published similar results using an αAstree electronic tongue, and PLSDA models showed high accuracy in recognition and prediction of pure honeys. In our study, linden honeys presented better results for our authentic honeys and were classified correctly. Our acacia results were similar in that 97.47% correct classification was found during calibration and 100% was found for prediction for pure honeys [40]. The results of the electronic nose showed a 94.34% correct classification for acacia and linden honey during training and 92.74% during validation, respectively. Our results are similar to those of another Chinese study using an electronic nose for the detection of adulteration of rape honey in 0–70% with rice syrup, where researchers could distinguish adulterated honeys from pure honeys [41]. Malaysian researchers also used the electronic nose for classification of honeys adulterated at the 20–80% levels. Their results showed clear separation of pure honeys, which is in line with our results [42]. In their another study, the electronic tongue and nose were used for the same purpose, resulting in 98.9% accuracy for the electronic nose and 96.7% after cross validation with LDA. These results are more accurate than ours. However, different types of honey and higher steps between adulteration levels were used [22]. Independent prediction of EUnonEU honey showed different results in the cases of ET and EN, yet HA_99 honey was classified as adulterated by both instruments (Table 5). This sample was also significantly different based on its reference parameters, such as CUPRAC, FRAP and pH compared to the authentic honey. HA_100 can also be suspected to be adulterated based on electronic tongue and nose results (Table 5). Moreover, sensory evaluation revealed significant differences for this honey in sweet and animalic odour and in sweet taste, flowery and caramel flavour (Figure 1). Similar results were obtained for HL_98 honey, which was significantly different based on the sensory parameters compared to the authentic honeys.

The prediction of the parameters of sensory profile analysis of the linden and acacia honeys provided better results (Table 6) in the case of linden honey for both instruments and can be explained by the lower aromatic properties of the acacia honey [36]. Moreover, better PLSR models were obtained using the data of the electronic tongue compared to the electronic nose. The better prediction in PLRS results in the case of linden honey for the bitter-related parameters such as bitter taste and medicinal flavour can be explained by the slightly bitter taste of linden honey, while the lower prediction abilities for sour taste could be the result of the weak acidity of the linden honey in general. The good results for flowery aroma of the acacia honey can be accounted for by the fact that acacia honey has a specific, pronounced flowery aroma [36]. Independent prediction of the sensory properties of EUnonEU honeys showed the most different results (Figure 4) for HA_99 honey, which was also classified as an adulterated honey by the electronic nose and tongue (Figure 2 and Figure 3). The sweeter taste of HL_79 and HL_83 honey can be explained by the higher sugar content compared to authentic honeys (Table 4).

## 5. Conclusions

Physicochemical evaluations revealed differences between authentic and adulterated honeys, showing that these simple methods are able to reveal some of the differences between adulterated and unadulterated honeys and samples of foreign origin. Sensory profile analysis showed that, in the case of acacia, the panel found significant differences between the reference and adulterated samples only in terms of four parameters and, in the case of linden honeys, in terms of eight parameters. These results show that, especially in the case of acacia honeys, humans are not able to detect the differences at lower adulteration levels. The electronic nose and tongue were far more sensitive in the discrimination of different honeys. In addition, the electronic nose and tongue provided promising results in revealing adulteration of honeys from EUnonEU regions using the results of the authentic and adulterated honey samples. The two instruments were both able to predict the sensory parameters at high accuracy in the case of linden honey, while the electronic tongue could predict the sensory properties of acacia honey better than the electronic nose. The study confirms the applicability of EN and ET for the detection of honey adulteration with sugar syrup; however, further studies are needed to build more robust models for more concentration levels of the adulterant using different types of syrups.

## Figures and Tables

**Table 1:** Botanical and geographical origins of authentic honey samples.

Sample Code	Botanical Origin	Geographical Origin	Altitude	Latitude	Longitude
HA_5	Acacia	Nyírbogát	150 m	47.8014742	22.0620214
HA_6	Acacia	Hajdúsámson	132 m	47.5989514	21.7537139
HA_7	Acacia	Jásszentandrás	100 m	47.58291768	20.17316437
HA_8	Acacia	Erdőtelek	107 m	47.6867102	20.3144529
HA_9	Acacia	Nyírség region *	127 m	47.9074163	22.0009761
HA_10	Acacia	Kisköre	87 m	47.4994568	20.4925043
HA_21	Acacia	Tura	120 m	47.60935	19.5949442
HA_29	Acacia	Salgótarján	239 m	48.0960676	19.8005642
HA_38	Acacia	Ősagárd	271 m	47.8578715	19.1953614
HA_63	Acacia	Kőtelek	84 m	47.3364243	20.4355722
HA_97	Acacia	Kisköre	87 m	47.4994568	20.4925043
HA_101	Acacia	Eger	169 m	47.8989887	20.3743665
HL_15	Linden	Kisköre	87 m	47.4994568	20.4925043
HL_16	Linden	Tiszanána	87 m	47.5564803	20.5292959
HL_17	Linden	Harghita region (RO) *	782 m	46.6440949	25.6200809
HL_35	Linden	Zselic	193 m	46.2030795	17.88148478
HL_43	Linden	Zalacsány	122 m	46.8065059	17.097903
HL_45	Linden	Covasna region (RO) *	566 m	45.8448991	26.1693108
HL_60	Linden	Kőtelek	84 m	47.3364243	20.4355722
HL_102	Linden	Eger	169 m	47.8989887	20.3743665
HL_103	Linden	Cegléd	106 m	47.1716447	19.7977516

* Only projections are given for regions. Source: https://www.mapcoordinates.net/en.

**Table 2:** Sensory properties of acacia and linden honeys defined by the sensory panel.

Acacia Characteristics	Linden Characteristics
odour intensity	odour intensity
flowery odour	resinous odour
fruity odour	medicinal odour
sweet odour	fresh odour
animalic odour	taste intensity
dry hay odour	sweet taste
taste intensity	bitter taste
sweet taste	sour taste
sour taste	resinous flavour
flowery flavour	medicinal flavour
caramel flavour	refreshing flavour
taste persistence	taste persistence
dry hay flavour	astringency

**Table 3:** Physicochemical, antioxidant and colour properties of acacia and linden honey types.

Acacia						Linden				
	Acacia	EUnonEU Acacia	A10	A20	A50	Linden	EUnonEU linden	L10	L20	L50
TPC mg GAE/100 g	4.92 ± 3.47 ^ab^	5.45 ± 1.46 ^a^	3.81 ± 0.27 ^b^	3.78 ± 0.45 ^bc^	2.40 ± 0.43 ^c^	9.68 ± 2.48 ^ab^	8.53 ± 1.23 ^bc^	10.2 ± 0.24 ^a^	10.09 ± 0.62 ^a^	7.55 ± 0.41 ^c^
CUPRAC µmol TEQ/g	12.32 ± 6.56 ^ab^	9.28 ± 3.19 ^c^	12.12 ± 0.35 ^a^	11.12 ± 0.33 ^b^	7.22 ± 20.34 ^d^	39.83 ± 14.86 ^a^	30.6 ± 5.16 ^b^	39.11 ± 1.51 ^a^	37.89 ± 0.96 ^a^	24.98 ± 0.53 ^c^
FRAP mg ASE/100 g	5.87 ± 3.17 ^a^	6.19 ± 4.08 ^a^	4.03 ± 0.09 ^b^	3.32 ± 0.18 ^c^	1.54 ± 0.19 ^d^	32.14 ± 13.14 ^a^	14.43 ± 3.95 ^b^	26.07 ± 1.21 ^c^	24.09 ± 1.39 ^c^	13.95 ± 0.37 ^b^
Total soluble dry matter %	81.8 ± 0.91 ^a^	82.78 ± 1.28 ^b^	80.1 ± 0.07 ^c^	79.4 ± 0 ^d^	77.4 ± 0 ^e^	82.11 ± 1.63 ^a^	81.67 ± 0.24 ^a^	81.9 ± 0.00 ^a^	81.4 ± 0.00 ^b^	78.7 ± 0.00 ^c^
pH	3.87 ± 0.20 ^a^	3.77 ± 0.16 ^b^	3.52 ± 0.02 ^c^	3.44 ± 0.00 ^d^	3.54 ± 0.04 ^c^	4.12 ± 0.20 ^a^	4.09 ± 0.07 ^a^	4.06 ± 0.01 ^a^	4.03 ± 0.01 ^b^	3.97 ± 0.01 ^d^
Electrical conductivity µS/cm	156.55 ± 26.15 ^a^	161.4 ± 29.52 ^a^	147 ± 0.71 ^b^	134.33 ± 0.41 ^c^	121.33 ± 0.41 ^d^	464.74 ± 137.38 ^a^	308.89 ± 75.71 ^b^	627.67 ± 1.47 ^c^	566 ± 0.71 ^d^	402.67 ± 1.08 ^e^
L*	58.5 ± 2.7^a^	56.51 ± 2.76 ^b^	60.14 ± 0.5 ^c^	60.33 ± 0.39 ^c^	60.31 ± 0.23 ^c^	51.19 ± 5.22 ^a^	51.6 ± 2.09 ^a^	55.13 ± 0.48 ^b^	55.67 ± 0.3 ^bc^	56.57 ± 0.48 ^c^
a*	−1.65 ± 0.81 ^bc^	−2.12 ± 0.32 ^a^	−1.71 ± 0.08 ^b^	−1.72 ± 0.1 ^b^	−1.4 ± 0.06 ^c^	1.54 ± 5.9 ^c^	−1.67 ± 1.49 ^ab^	−2.03 ± 0.07 ^b^	−2.15 ± 0.02 ^b^	−2.75 ± 0.09 ^a^
b*	13.49 ± 7.15 ^a^	15.21 ± 2.78 ^a^	9.09 ± 0.03 ^b^	9.92 ± 0.06 ^c^	7.23 ± 0.09 ^d^	31.31 ± 8.91 ^b^	23.9 ± 3.66 ^a^	28.14 ± 0.54 ^b^	28.2 ± 0.12 ^b^	24.6 ± 0.26 ^a^
Glucose g/kg	252.87 ± 17.52 ^a^	275.74 ± 18.95 ^b^	-	-	-	286.04 ± 26.30 ^a^	345.63 ± 37.14 ^b^	-	-	-
Fructose g/kg	417.61 ± 12.66 ^a^	443.26 ± 57.26 ^b^	-	-	-	389.27 ± 22.43 ^a^	410.26 ± 16.54 ^b^	-	-	-

Mean ± standard deviation. Letters denote the significant differences between groups based on results of ANOVA followed by the Games–Howell pairwise comparison separately for acacia and linden honey types. Samples with no results were not analysed for the sugar composition.

**Table 4:** Significant differences of blends of honeys of both European and non-European Union origins in physicochemical and colour results for linden and acacia honeys.

	TPC mg GAE/100 g N = 5/Sample	CUPRAC µmol TEQ/g N = 5/Sample	FRAP mg ASE/100 g n = 5/Sample	Total Soluble Dry Matter % n = 3/Sample	pH n = 3/Sample	Electrical Conductivity µS/cm n = 3/Sample	Glucose g/kg n = 2/Sample	Fructose g/kg n = 2/Sample
**Authentic Acacia**	**4.92 ± 3.47**	**12.32 ± 6.56**	**5.87 ± 3.17**	**81.8 ± 0.91**	**3.87 ± 0.2**	**156.55 ± 26.15**	**252.87 ± 17.52**	**417.61 ± 12.66**
HA_100	4.37 ± 0.67	5.68 ± 0.42 ***	1.35 ± 0.21 ***	85 ± 0.14 ***	3.52 ± 0.01 ***	158.67 ± 0.41	312.41 ± 2.27 ***	503.42 ± 0.88 ***
HA_78	6.93 ± 1.26	9.30 ± 0.72	8.16 ± 0.24 ***	81.4 ± 0 *	3.73 ± 0 ***	116.00 ± 0 ***	275.90 ± 6.34 **	476.35 ± 2.74 ***
HA_84	6.01 ± 0.42	11.2 ± 1.66	7.40 ± 3.12	84.2 ± 0 ***	3.84 ± 0	175.00 ± 0 ***	271.00 ± 8.16	409.27 ± 13.94
HA_99	4.94 ± 1.20	5.68 ± 1.39 ***	2.40 ± 0.96 **	81.8 ± 0	3.76 ± 0 **	173.67 ± 0.41 ***	NA	NA
**Authentic Linden**	**9.68 ± 2.48**	**39.83 ± 14.86**	**32.14 ± 13.14**	**82.11 ± 1.63**	**4.12 ± 0.2**	**464.74 ± 137.38**	**286.04 ± 26.30**	**389.27 ± 22.43**
HL_79	9.44 ± 1.14	37.02 ± 2.64	17.98 ± 1.55 ***	81.8 ± 0	4.17 ± 0	388.67 ± 0.41 **	310.49 ± 1.86 ***	394.81 ± 0.25
HL_83	7.41 ± 1.11	28.72 ± 1.7 ***	12.34 ± 5.31 ***	81.6 ± 0.28	4.03 ± 0.02 *	212 ± 1.41 ***	380.77 ± 3.56 ***	425.72 ± 4.25 ***
HL_98	8.74 ± 0.4	26.05 ± 1.23 ***	12.97 ± 0.29 ***	81.6 ± 0.28	4.05 ± 0.01	326 ± 1.22 ***	NA	NA

Mean ± standard deviation. The asterisk * denotes the significant differences from authentic honeys based on the results of ANOVA test followed by the pairwise comparison: * *p* < 0.05, ** *p* < 0.01 and *** *p* < 0.001.

**Table 5:** Classification results of independent prediction of EUnonEU acacia and linden honeys.

	Electronic Tongue				Electronic Nose			
	Authentic	10% Syrup	20% Syrup	50% Syrup	Authentic	10% Syrup	20% Syrup	50% Syrup
HA_100	88.24%	11.76%	0.00%	0.00%	40.74%	59.26%	0.00%	0.00%
HA_78	96.15%	3.85%	0.00%	0.00%	5.26%	0.00%	94.74%	0.00%
HA_84	100.00%	0.00%	0.00%	0.00%	29.63%	70.37%	0.00%	0.00%
HA_99	0.00%	33.33%	0.00%	66.67%	0.00%	16.67%	83.33%	0.00%
HL_79	100.00%	0.00%	0.00%	0.00%	0.00%	0.00%	0.00%	100.00%
HL_83	100.00%	0.00%	0.00%	0.00%	29.63%	29.63%	22.22%	18.52%
HL_98	0.00%	0.00%	0.00%	100.00%	52.38%	38.10%	9.52%	0.00%

HA means acacia, HL means linden, and numbers are the registered numbers of the honeys.

**Table 6:** Results of the regression models built on the properties of sensory profile analyses of acacia and linden honey used in sensory profile analyses based on data of the electronic tongue and nose.

		**Electronic Tongue**						**Electronic Nose**					
		**Latent variables**	**Data points**	**R^2^**	**R^2^CV**	**RMSE**	**RMSECV**	**Latent variables**	**Data points**	**R^2^**	**R^2^CV**	**RMSE**	**RMSECV**
**Acacia**	**fruity_odour**	4	59	0.7966	0.7520	2.6617	2.9360	NA	NA	NA	NA	NA	NA
	**animalic_odour**	5	60	0.9541	0.9415	3.6172	4.0789	4	38	0.5715	0.3321	10.5446	13.1686
	**flowery_odour**	5	59	0.9102	0.8826	2.5253	2.8853	4	37	0.6334	0.4083	5.0010	6.3486
	**fresh_odour**	6	60	0.8972	0.8688	2.1964	2.4790	4	42	0.3331	0.0224	5.3930	6.5175
	**flowery_flavour**	5	53	0.9147	0.8880	3.5391	4.0509	4	38	0.6526	0.4564	6.8022	8.5140
	**sweet_taste**	3	63	0.6049	0.5244	4.2802	4.6922	4	40	0.6385	0.4578	3.9798	4.8696
	**caramel_flavour**	6	58	0.7467	0.6765	5.2581	5.9363	4	40	0.5621	0.3060	7.1048	8.9776
		**Electronic tongue**						**Electronic nose**					
		**Latent variables**	**Data points**	**R^2^**	**R^2^CV**	**RMSE**	**RMSECV**	**Latent variables**	**Data points**	**R^2^**	**R^2^CV**	**RMSE**	**RMSECV**
**Linden**	**odour_intensity**	4	43	0.9732	0.9666	2.2630	2.5251	4	35	0.8314	0.7290	4.7812	6.0297
	**resinous_odour**	5	46	0.9534	0.9399	1.7048	1.9322	3	32	0.8681	0.8126	2.7420	3.2589
	**fresh_odour**	4	41	0.8404	0.7964	2.6335	2.9707	3	31	0.5388	0.3851	3.7529	4.3241
	**taste_intensity**	4	44	0.8670	0.8357	2.7052	3.0034	3	32	0.7987	0.7244	3.3300	3.8900
	**bitter_taste**	4	43	0.9128	0.8882	2.2107	2.5010	3	32	0.9362	0.9044	1.4488	1.7662
	**sour_taste**	3	44	0.8331	0.8039	2.0882	2.2615	4	37	0.5722	0.3690	3.3499	4.0562
	**sweet_taste**	4	45	0.8658	0.8204	3.1057	3.5876	3	32	0.8800	0.8121	2.5577	3.1829
	**medicinal_flavour**	4	42	0.9142	0.8884	2.7856	3.1724	4	35	0.9288	0.9011	2.3415	2.7549
	**refreshing_flavour**	4	41	0.8624	0.8235	2.5896	2.9287	3	34	0.5392	0.3840	4.1009	4.7324
	**taste_persistence**	4	42	0.9511	0.9399	2.3533	2.6051	3	33	0.8706	0.8274	3.9727	4.5988

Parameters showing significant differences among the honeys in sensory profile analyses were chosen to build PLRS models.
